# Appropriate Amounts and Activity of the Wilms’ Tumor Suppressor Gene, *wt1*, Are Required for Normal Pronephros Development of *Xenopus* Embryos

**DOI:** 10.3390/jdb10040046

**Published:** 2022-10-29

**Authors:** Taisei Shiraki, Takuma Hayashi, Jotaro Ozue, Minoru Watanabe

**Affiliations:** 1 Graduate School of Sciences and Technology for Innovation, Tokushima University, 1-1 Minamijosanjima-Cho, Tokushima 770-8054, Japan; 2 Institute of Liberal Arts and Sciences, Tokushima University, 1-1 Minamijosanjima-Cho, Tokushima 770-8054, Japan

**Keywords:** CRISPR/Cas9, pronephros, transcription factor, Wilms’ tumor, WT1, *Xenopus*

## Abstract

The Wilms’ tumor suppressor gene, *wt1*, encodes a zinc finger-containing transcription factor that binds to a GC-rich motif and regulates the transcription of target genes. *wt1* was first identified as a tumor suppressor gene in Wilms’ tumor, a pediatric kidney tumor, and has been implicated in normal kidney development. The WT1 protein has transcriptional activation and repression domains and acts as a transcriptional activator or repressor, depending on the target gene and context. In *Xenopus*, an ortholog of *wt1* has been isolated and shown to be expressed in the developing embryonic pronephros. To investigate the role of *wt1* in pronephros development in *Xenopus* embryos, we mutated *wt1* by CRISPR/Cas9 and found that the expression of pronephros marker genes was reduced. In reporter assays in which known WT1 binding sequences were placed upstream of the *luciferase* gene, WT1 activated transcription of the *luciferase* gene. The injection of wild-type or artificially altered transcriptional activity of *wt1* mRNA disrupted the expression of pronephros marker genes in the embryos. These results suggest that the appropriate amounts and activity of WT1 protein are required for normal pronephros development in *Xenopus* embryos.

## 1. Introduction

*wt1* was identified by positional cloning as a responsible gene for Wilms’ tumor, a pediatric kidney tumor that affects 1 in 10,000 children [1,2,3]. Since deletions or mutations of this gene were found in approximately 15% of Wilms’ tumors, this gene has been thought to be a tumor suppressor gene [4]. However, in other Wilms’ tumors, *wt1* is expressed at higher levels than normal, suggesting that only a small fraction of Wilms’ tumors are caused by loss-of-function of *wt1* [5,6]. Other cancers, such as leukemia and breast cancer, have been shown to overexpress *wt1*, making this gene an oncogene [6,7]. Currently, the functions of *wt1* in cancers are thought to depend on the cell and tissue types. *wt1* is expressed in developing kidneys [8,9,10], and targeted mutation of *wt1* caused a failure of kidney development in mice embryos [11]. In mouse kidney explant assays, morpholino antisense oligo to *wt1* inhibited kidney development [12]. These results strongly suggest that *wt1* plays an essential role in mammalian kidney development.

*wt1* encodes a protein with four C_2_H_2_ zinc finger motifs at its C-terminus, similar to the SP1 family of transcription factors [1,2,3]. These C_2_H_2_ motifs have been shown to bind to the GC-rich motif, suggesting that the WT1 protein acts as a transcription factor [13,14]. Indeed, the transcriptional activity of WT1 has been extensively studied, and the N-terminal region of this protein has been found to contain transcriptional activator and repressor domains that, depending on the target gene and context, act as transcriptional activators in some cases and repressors in others [14,15]. In addition, more than 20 splicing variants exist in the mammalian *wt1* gene [16]. Among these splicing variants, the insertion of 17 amino acids (17aa) after the activation domain and three amino acids (lysine, threonine, and serin; KTS) between the third and fourth zinc finger domains are conserved in mammals [16]. Interestingly, while KTS insertion has been found in all animals, the 17aa insertion has only been identified in mammals, suggesting an evolutionary significance of the 17aa insertion in mammals [16]. Insertion of the KTS within the zinc finger DNA binding domain results in the reduction of DNA-binding activity, and +KTS variants have been shown to act as RNA-binding factors [17]. This context-dependent transcriptional activity and the many variants with distinct functions make it very difficult to understand the nature of WT1.

The *Xenopus* ortholog of *wt1* has been isolated and shown to be expressed in the developing pronephros, an embryonic kidney of *Xenopus* embryos [18,19]. It has also been found that overexpression of *wt1* in the embryos suppressed pronephros-specific *xlim-1* gene expression, which is essential for normal pronephros development [20,21]. These results indicate that in *Xenopus*, as in mammals, *wt1* plays an essential role in pronephros development. In this research, to investigate the function of *wt1* in pronephros development further, we mutated *wt1* using the CRISPR/Cas9 method. We found that the expression of pronephros marker genes was reduced in these embryos. We also found that in reporter assays in which known WT1 binding sequences were placed upstream of the *luciferase* gene, WT1 activated transcription of the *luciferase* gene. Injection of the *wt1* mRNA of wild-type or artificially altered transcriptional activity disrupted the expression of pronephros marker genes. These results suggest that the appropriate amounts and activity of the WT1 protein are required for normal pronephros development in *Xenopus* embryos.

## 2. Materials and Methods

### 2.1. Embryos and Microinjection

Eggs from female frogs injected with the ovulation-inducing hormone (hCG, human chorionic gonadotropin, 700 units/frog, ASKA Pharmaceutical Co., Ltd. Tokyo, Japan) were artificially fertilized using testis homogenate. They were dejellied with 3% cysteine (pH 7.8) followed by several washes with 0.1 × MMR (1 × MMR: 100 mM NaCl, 2 mM KCl, 2 mM CaCl_2_, 1 mM MgCl_2_, 5 mM HEPES [pH 7.4]), 30 min after fertilization. Embryos were staged, according to Nieuwkoop and Faber [22]. Microinjection was done to both cells animally at the 2-cell stage for *wt1*-sgRNA/Cas9, *tyrosinase*-sgRNA/Cas9 or *luciferase* reporter experiments, with 10 nl volume per injection in 1 × MMR with 3% Ficoll 400. For the *wt1* mRNAs, a single vegetal ventral cell injection on the right side at the 16-cell stage embryos was done to target cells that give rise to pronephros. Microinjection was performed under a microscope with a Mk1 micromanipulator (Singer Instruments, Somerset, UK) and a IM 300 microinjector (Narishige, Tokyo, Japan).

### 2.2. Whole-Mount In Situ Hybridization (WISH)

WISH was performed essentially as described [23], except RNase treatment was omitted, and BM purple (Roche Diagnostics, Tokyo, Japan) was used as a substrate. Antisense *xlim-1* or *pax2* template was linearized with *Xho*I and transcribed with T7 RNA polymerase or *Eco*RI and T3 RNA polymerase, respectively, using digoxigenin-labeled UTP (Roche Diagnostics, Tokyo, Japan).

### 2.3. Beta-Gal Staining

Embryos injected with mRNA encoding nuclear beta-galactosidase (100 pg/embryo) were fixed by MEMFA (0.1 M MOPS (pH 7.4), 2 mM EGTA, 1 mM MgSO_4_ and 3.7% formaldehyde) for 15 min, washed by PBS containing 2 mM MgCl_2_ and stained in PBS containing 1 mg/mL X-gal, 20 mM K_3_Fe(CN)_6_, 20 mM K_4_Fe(CN)_6_, 2 mM MgCl_2_ for 1 h at room temperature.

### 2.4. CRISPR/Cas9 Knockout of wt1 and tyrosinase

**Figure 1:**
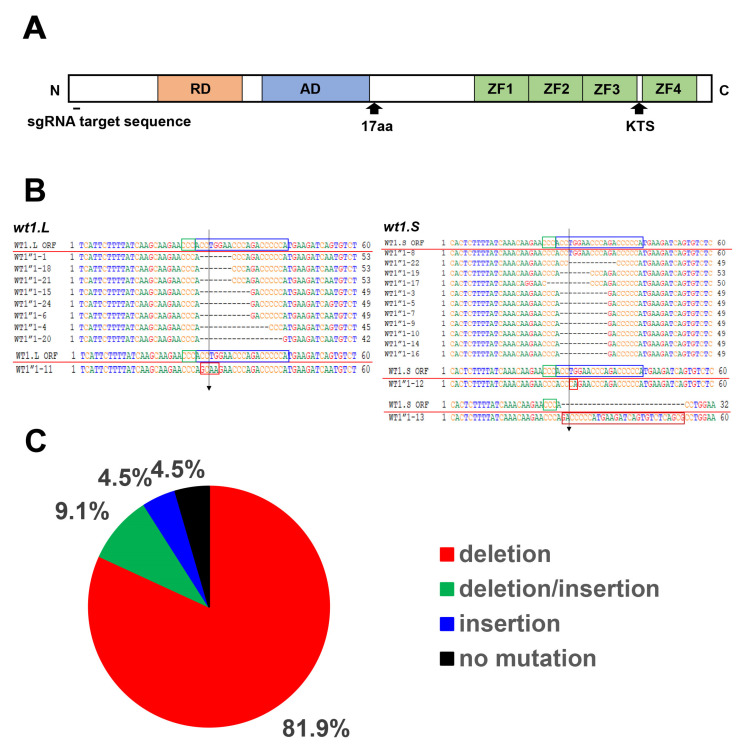
Schematic diagram of the WT1 protein and the results of CRISPR/Cas9 knockout of *wt1*. (**A**) Schematic of the WT1 protein. 17aa and KTS represent splicing variants with or without these insertions (note that 17aa insertion variants are present only in mammals). The target sequence of sgRNA is in the N-terminus. Abbreviations are as follows; RD; transcription repression domain, AD transcription activation domain, ZF; zinc finger DNA binding domain. (**B**) Details of mutations in *wt1* caused by the injection of *wt1-*sgRNA/Cas9. Mutations in the *wt1.L* gene are shown on the left and in the *wt1.S* gene on the right. The sequence of wild-type *wt1* is shown above the red line. Blue squares indicate sgRNA target sequences, and green squares indicate PAM sequences. Vertical arrows indicate the positions where the Cas9 protein is thought to cleave DNA. Deleted nucleotides are indicated by dots, replaced nucleotides by red squares, and inserted nucleotides by brown square. Each sequence represents an independent DNA clone. (**C**) Summary of mutations results from CRISPR/Cas9 method. A total of 22 DNA clones were sequenced, 10 from the *wt1.L* gene and 12 from the *wt1*.*S* gene. The graph summarizes the mutations of both homeologs.

For the sgRNA target sequence, we selected 20 nucleotides near the N-terminus of the WT1 protein upstream of the PAM sequence (5′ NGG 3′), where *wt1.L* and *wt1.S* sequences were identical. The reason for setting the target sequence near the N-terminus of the WT1 protein was the expectation that the frameshift mutation would result in loss of protein function. After selecting target sequences, sgRNA templates were prepared by PCR-based methods. sgRNA plasmid DR274 (Addgene, MA, USA), which was *Dra*I-digested, and 5′ *TAATACGACTCACTATA*NNNNNNNNNNNNNNNNNNNNGTTTTAGAGCTAGAAATAGC 3′ and 5′ AAAAGCACCGACTCGTGCC 3′ (T7 promoter sequence in italics, Cas9 protein binding sequence of DR274 in underline, N-stretch is each target sequence) were used to create sgRNA templates by PCR. The resulting PCR products were 117 bp long, including the T7 promoter (17 bp), target sequence (20 bp), and Cas9 protein binding sequence (80 bp), in order from 5′ to 3′. These DNAs were subjected to T7 RNA transcriptase reaction to synthesize each sgRNA. The target sequence of the *wt1*-sgRNA was 5′ TGGGGGTCTGGGTTCCAGGT 3′, which is an antisense strand of *wt1* (Figure 1B). Each embryo was injected with a mixture of 1.3 ng of *wt1*-sgRNA and 6.44 ng of Cas9 protein (EnGen Cas9 NLS, S. pyogenes, NEB, MA, USA) to approximately the same molar concentration. The *wt1*-sgRNA/Cas9 was injected in both cells at the 2-cell stage, so half the amount of the sgRNA and Cas9 protein mixture was injected in each cell. Five sgRNA/Cas9 injected embryos (stage 20) were collected in one tube from which DNA was extracted. Therefore, the DNA was a mixture of the five embryos. Genomic DNA containing the target sequence was then PCR amplified by using a pair of primers, 5′ TCAGTGGGCTCCAGTCCTGGACTT 3′ and 5′ GAACATCCTTGCTTGGCCTGTTGT 3′. Amplified DNAs were subcloned into T-vector (pGEM-T easy, Promega, WI, USA), and nucleotide sequences were determined by Eurofinsgenomics, Inc. (Tokyo, Japan). A total of 22 DNA clones were sequenced, 10 from *wt1.L* and 12 from *wt1.S*. The target sequence of the *tyrosinase*-sgRNA was 5′ GGCCCACTGCTCAGAAACCC 3′, identical in both *tyrosinase. L.* and *S*. Synthesis of *tyrosinase*-sgRNA was essentially identical to that used for *wt1-*sgRNA.

### 2.5. Plasmid Construction and mRNA Synthesis

*wild-type wt1*: total RNA from a male frog kidney was extracted, and cDNA was synthesized using oligo (dT) primer (Oligo(dT)20 Primer, Toyobo, Osaka, Japan) and reverse transcriptase (PrimeScript Reverse Transcriptase, Takara Bio, Tokyo, Japan). Using this cDNA as a template, WT1.L protein coding sequence was PCR amplified using the following primer sets; 5′ GGCTCGAGATGGGATCTGATGTGCGG 3′ (the underline represents *Xho*I site for subcloning) and 5′ GGGCTAGCCTAAAGGGCCAGATGGAGTT 3′ (the underline represents *Nhe*I site for subcloning), and subcloned into the *Xho*I/*Nh*eI digested pCS4-3HA vector. The nucleotide sequence of the resultant plasmid was confirmed by sequencing. This construct was linearized by *Not*I, and mRNA was transcribed by SP6 RNA polymerase (mMESSAGE mMACHINE™ SP6 Transcription Kit, Thermo Fisher Scientific, MA, USA).

*vp16-wt1* and *en-wt1*: the four-zinc finger DNA binding domain sequence from the wild-type *wt1* gene was PCR amplified using the following primer set; 5′ GGCTCGAGAGAGGAATTCAAGATGTGAG 3′ (the underline represents *Xho*I site for subcloning) and 5′ GGGCTAGCCTAAAGGGCCAGCTGGAGAA 3′ (the underline represents *Nhe*I site for subcloning). The PCR products were subcloned into *Xho*I/*Nhe*I digested pCS4-HA-VP16 or pCS4-3HA-En vector. Synthesis of mRNA from these constructs was performed in the same manner as described above.

### 2.6. Luciferase Reporter Assay

A canonical WT1 binding sequence and TATA box containing oligonucleotide pair, 5′ GATCCGAGTGCGGGGGCGAGAATT*AGGGTATATAATG* 3′ and 5′ AGCT*CATTATATACCCT*AATTCTCGCCCCCGCACTCG 3′ (the underlines represent the WT1 binding sequences, and the italics represent TATA box from adenovirus type 5 early region 4 promotor), was annealed and inserted into *Bgl*II/*Hin*dIII digested *luciferase* vector (pGL3-Basic vector, Promega, WI, USA). The nucleotide sequence of the resultant reporter construct was confirmed by sequencing. Another WT1-binding sequence, 5′ GCGTGGGAGT 3′, containing *luciferase* reporter was synthesized in the same way. Luciferase reporter (100 pg/embryo) was injected with or without *wt1* mRNA (500 pg/embryo) into 2-cell stage embryos at the animal pole. Four injected embryos were collected in each tube, and 4–5 tubes per injection were prepared. The mean and standard deviation were calculated from the Luciferase activity of these 4–5 tubes. The Luciferase activity was measured using the Luciferase Reporter Assay System (Promega, WI, USA)

## 3. Results

### 3.1. Reduced Expression of the Pronephros Marker Genes in the wt1 Knockout Embryos by CRISPR/Cas9 Method

To investigate the role of *wt1* in the pronephros development of *Xenopus* embryos, we attempted to knockout *wt1* by the CRISPR/Cas9 method. Because *Xenopus laevis* is an allotetraploid species, most genes have two homeologs, *L* and *S*, and *wt1* had these alleles [24,25]. The sgRNA target sequence of *wt1* which encodes a protein and is identical to the *wt1.L* and *wt1.S* homeologs, was selected, and correspondence sgRNA was synthesized (Figure 1A). The *wt1*-sgRNA was injected into the 2-cell stage embryos together with Cas9 protein, and injected embryos were allowed to develop. When the embryos reached the neurula stage (stage 20), genomic DNAs were extracted, the DNA sequences containing the sgRNA target sequence were amplified by PCR, and the nucleotide sequences were determined after subcloning to a plasmid vector. Mutations were observed in 21 of the 22 clones sequenced (Figure 1B,C). As shown in Figure 1B,C, most of the mutations were deletions (81.1%), while others were insertions (4.5%) or replacements to other sequences (9.1%). Mutations were found in both *wt1.L* (*n* = 10) and *wt1.S* (*n* = 12) genes, suggesting that the identical sgRNA target sequences in both homeologs are efficiently mutated (Figure 1B,C). However, 4 of the 21 mutated sequences were found to be in-frame mutations, which may not affect WT1 protein function (Figure 1B). Nevertheless, these results indicate that the CRISPR/Cas9 method efficiently introduces mutations in *wt1*.

**Figure 2:**
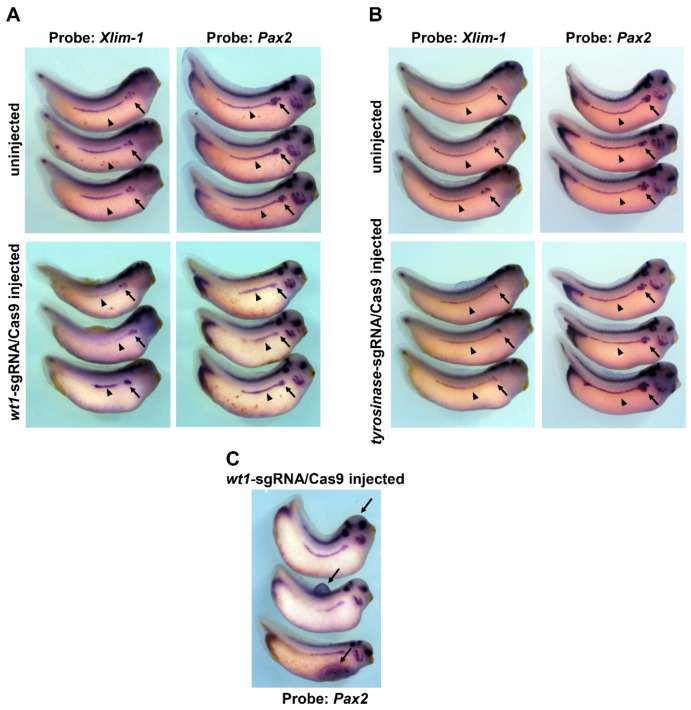
Expression of the *xlim-1* and *pax2* genes in embryos injected with *wt1*-sgRNA/Cas9. (**A**) Expression of the *xlim-1* and *pax2* genes in embryos injected with *wt1*-sgRNA/Cas9 are shown with representative embryos and probe combinations of results. Arrows indicate expression in the pronephric tubules, and arrowheads indicate expression in the pronephric ducts. (**B**) Expression of the *xlim-1* and *pax2* genes in embryos injected with *tyrosinase*-sgRNA/Cas9 are shown with representative embryos and probe combinations of results. Arrows and arrowheads indicate the same as in (**A**)**.** (**C**) Edema induced in embryos by injection of *wt1*-sgRNA/Cas9. These embryos also show the expression of the *pax2* gene. The top embryo has edema in the head, the middle embryo in the back, and the bottom embryo in the abdomen (the abdominal edema is collapsed). Arrows indicate the regions edema occurred. Note that the morphology of the embryo is altered due to the development of edema.

Next, we examined the development of pronephros in *wt1* knockout embryos. To investigate the pronephros development, we examined the expression of the *xlim-1* and *pax2* genes, which are expressed in the developing pronephros and play an essential role in pronephros development [21,26,27,28]. When *wt1-*sgRNA/Cas9 injected embryos reached stage 34/35, the stage functional pronephros is formed, the expression of the *xlim-1* and *pax2* genes were examined by whole-mount *in situ* hybridization (WISH). As shown in Figure 2A, the *xlim-1* and *pax2* genes were expressed in pronephric tubules (arrows) and ducts (arrowheads) in uninjected embryos. In contrast, expression of *xlim-1* (49%, *n* = 35) or *pax2* (50%, *n* = 36) was reduced in the embryos injected with *wt1*-sgRNA/Cas9 (arrows and arrowheads in Figure 2A). As a control experiment, *tyrosinase*-sgRNA/Cas9 injection was performed. However, no differences in pronephros marker gene expression were observed between control and experimental embryos (Figure 2B). Edema was also observed in some of the injected embryos (Figure 2C). These results suggest that the normal pronephros development in embryos injected with *wt1*-sgRNA/Cas9 was impaired.

### 3.2. Transcriptional Activity of the WT1 in Xenopus Embryo

**Figure 3:**
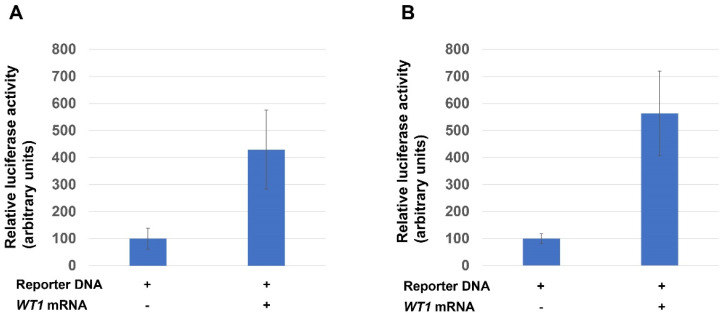
Activation of *luciferase* reporter DNA by the WT1. (**A**) Luciferase activity was examined in embryos by injecting reporter DNA (100 pg/embryos) containing canonical WT1-binding sequence (5′ GCGGGGGCG 3′) with or without *wt1* mRNA (500 pg/embryos) into the 2-cell stage embryos. The Luciferase activity was examined in the gastrula stage embryos (stage 11). The Luciferase activity of embryos without *wt1* mRNA injection was set at 100, and the Luciferase activity of embryos injected with *wt1* mRNA was expressed as a relative value. Error bars represent the standard deviations. Experiments were repeated at least three times, and the representative result is shown. (**B**) Another WT1 binding sequence (5′ GCGTGGGAGT 3′) was injected as in (**A**), and the representative result is shown.

Since knockout of *wt1* reduced the expression of pronephros markers, we next wanted to examine the effect of overexpression of this gene in pronephros development. The protein-coding sequence of the *wt1.L* gene was PCR amplified from adult *Xenopus* kidney cDNA, and the nucleotide sequences were determined [24,25]. As previously reported [18,19], two splicing variants were found, one contained the KST sequence between the third and fourth zinc finger DNA binding domains (+KTS), and the other did not (-KTS, See, Figure 1A). A variant with the 17 amino acids (17aa) insertion in N-terminus was not found in our experiment, as in previous reports [18,19]. This study focused on the -KTS variant since it has higher DNA-binding activity than +KTS [13,17,29]. The WT1 protein has transcriptional activation and repression domains in its N-terminus (see, Figure 1A). It is known that WT1 can activate or repress the expression of target genes. To examine the transcriptional activity in the early *Xenopus* embryos, we made a *luciferase* reporter construct containing canonical WT1-binding sequence, 5′ GCGGGGGCG 3′ (see, Materials and Method, [13]). This reporter DNA was injected with or without *wt1* mRNA, and Luciferase activity was examined at the gastrula stage (stage 11). As shown in Figure 3A, Luciferase activity was increased when *wt1* mRNA was injected (Figure 3A). We also made another reporter construct containing a WT1-binding sequence, 5′ GCGTGGGAGT 3′ with a higher binding affinity to the WT1 [29] and examined the transcriptional activity of the WT1. Again, injection of *wt1* mRNA increased Luciferase activity (Figure 3B). These results indicate that, at least in this experimental system, WT1 acts as a transcriptional activator.

### 3.3. Pronephric Phenotypes of the wt1 mRNA Injected Embryos

**Figure 4:**
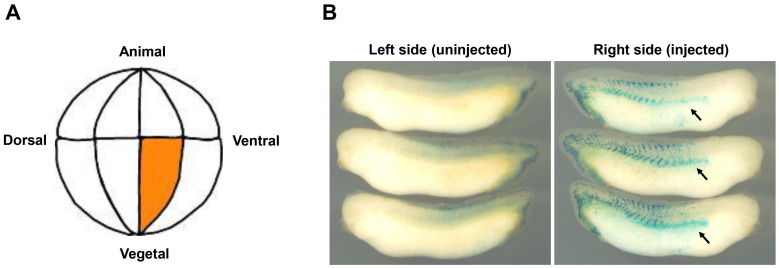

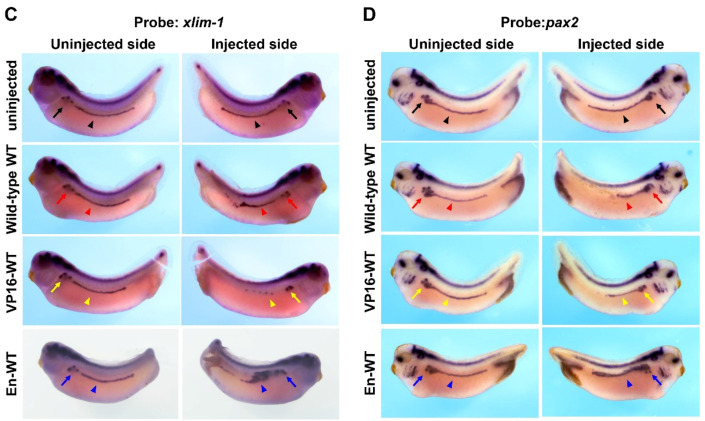
The *xlim-1* and *pax2* gene expression in embryos injected with wild-type, activator, or repressor form of *wt1* mRNA. (**A**) Schematic representation of the right-side view of the 16-cell stage embryo. An injected cell at the vegetal ventral side of the embryo, known to give rise to pronephros at a later stage, was orange-colored. (**B**) To confirm that the injected cell differentiates into pronephros, mRNA encoding nuclear beta-galactosidase was injected into the cell shown in (**A**), and the progenies of the injected cell were visualized at stage 34/35. The arrows indicate where the pronephros is formed. (**C**) Expression of the *xlim-1* gene in *wt1-*mRNA injected embryos. *wt1* mRNA of wild-type (500 pg/embryo), activator (100 pg/embryo), or repressor form (200 pg/embryo) was injected into a single cell at the 16-cell stage embryos as indicated in (**A**). Arrows indicate expression in the pronephric tubules, and arrowheads indicate expression in the pronephric ducts. (**D**) Expression of the *pax2* gene was examined as in (**C**).

We next examined the pronephros development of embryos injected with *wt1* mRNA. When wild-type *wt1* mRNA was injected at one cell of the 2-cell stage embryos, injected embryos showed abnormalities such as gastrulation defects and body curvature, which may be non-specific effects due to the overexpression of WT1 (data not shown). To avoid such non-specific effects, the *wt1* mRNA was injected into a single vegetal ventral cell on the right side of the 16-cell stage embryos, which is known to give rise to pronephros at a later stage (Figure 4A, [21,30,31]). To confirm that the injected cell differentiates into pronephros, mRNA encoding nuclear beta-galactosidase (nLacZ) injection was performed as a lineage tracer, and the progenies of the injected cell were visualized at stage 34/35. As shown in Figure 4B, most of the injected embryos (92.5%, *n* = 40) showed nLacZ staining in the region containing pronephros, indicating that the injection was correctly performed (Figure 4B). When such targeted injection of the *wt1* mRNA was performed, non-specific defects were considerably suppressed, although still edema phenotype was observed (data not shown). When injected embryos reached stage 34/35, embryos were harvested, and the expression of the *xlim-1* and *pax2* was studied. As shown in Figure 4C,D, the expression of both markers was reduced on the injected side compared to the uninjected side of the same embryo (*xlim-1* (52%, *n* = 23) and *pax2* (50%, *n* = 24), red arrows and arrowheads in Figure 4C,D), as previously reported [20]. These results suggest that overexpression of the WT1 inhibits normal pronephros development in the *Xenopus* embryos.

Since the WT1 was found to be a transcriptional activator, we attempted to alter the transcriptional activity of the WT1. Therefore, a potent transcriptional activation (VP16) or repression (En) domain was fused to WT1 with endogenous transcription domains removed (see, Figure 1A and Materials and Methods, [32]). These mRNAs were injected into a single vegetal ventral cell at the 16-cell stage embryos as wild-type *wt1* mRNA injection. When injected embryos reached stage34/35, the expression of pronephros marker genes was examined. As shown in Figure 4C,D, *vp16-wt1* mRNA injection reduced the expression of *xlim-1* (52%, *n* = 25) and *pax2* (50%, *n* = 28), as did wild-type *wt1* mRNA injection (yellow arrows and arrowheads in Figure 4C,D). In some injected embryos, the expression regions were not contiguous but interrupted (data not shown). Injection of the repressor form of WT1, En-WT1, caused disrupted expression of pronephros markers genes. In many cases, the expression of *xlim-1* (72%, *n* = 18) and *pax2* (47%, *n* = 17) were expanded (blue arrows and arrowheads in Figure 4C,D). In some injected embryos, the expression regions were widened, but the length of the regions was shortened (data not shown). These results suggest that altered transcriptional activities of the WT1 inhibit normal pronephros development of *Xenopus* embryos.

## 4. Discussion

*wt1* was identified as a tumor suppressor gene in Wilms’ tumor, and approximately 15% of Wilms’ tumors have mutations in *wt1* [1,2,3,4]. However, in other Wilms’ tumors, *wt1* is expressed normally, sometimes even more [5,6]. In mice, *wt1* is expressed in the developing kidney of the embryos, and targeted knockout of *wt1* resulted in embryonic lethality with failure of kidney development [11]. In addition, morpholino antisense oligo to *wt1* inhibited kidney development in mouse explant assays [12]. These results indicate that *wt1* is required for normal kidney development in mice. Knockout of *Xenopus wt1* with CRISPR/Cas9 also reduced the pronephros marker gene expression (Figure 2A), consistent with the results of mouse experiments. In embryos injected with CRISPR/Cas9, edema was observed (Figure 2C). Since edema is caused by excess fluid retention, pronephros function may be impaired in these embryos [33,34], as evidenced by reduced expression regions of marker genes, although it is caused by reasons other than kidney malfunction. In early *Xenopus* embryos, *wt1* is expressed in the anterior part of the pronephros, glomus anlage, not in tubules or duct anlages at the tailbud stage [18,28]. CRISPR/Cas9 knockout affected the pronephros marker expression in pronephric tubules and ducts, where *wt1* is not expressed. Since pronephric anlages are formed from anterior to posterior fashion during embryonic development [20,28,35], *wt1* might be involved in the early pronephros-inducing signaling pathway.

The transcriptional activity of *wt1* has been extensively studied [14,15,16,36,37]. *wt1* has been shown to repress many target genes, including the *pax2* gene, in mouse kidney development [38]. *wt1* also has been shown to activate the expression of target genes, and both repression and activation domains were mapped in WT1 (see, Figure 1A, [14,15]). Furthermore, chromatin immunoprecipitation (ChIP) analysis using mouse embryonic kidney tissue revealed more than thousands of potential WT1 target genes [12]. We studied the transcriptional activities of the WT1 in early *Xenopus* embryos using a simple *luciferase* reporter system containing WT1-binding sequences; we used two different but related binding sequences [14,29] and found that the WT1 activated the transcription from both sequences (Figure 3A,B). It is important to note that in artificial transcriptional assay systems, including this system, even the choice of expression vectors that drive the transcription of *wt1* may affect the transcriptional activity of the WT1 [39]. Therefore, care should be taken in interpreting the results of the transcriptional activities. However, the expression of pronephros markers in embryos injected with wild-type *wt1* mRNA was similar to those in embryos injected with *vp16-wt1* mRNA, suggesting that the WT1 acted as an activator in *Xenopus* embryos to some extent.

In wild-type *wt1* mRNA-injected embryos, as in our knockout experiments, we observed reduced expression of the *xlim-1* and *pax2* genes (red arrows and arrowheads in Figure 4C,D), as previously reported [20]. Why do overexpression and underexpression of the WT1 result in similar phenotypes? One possibility is that pronephros development in *Xenopus* is sensitive to the amount of WT1 protein and that normal pronephros development requires tight control of the amount of WT1 protein. Therefore, it is thought that changes in the amount of WT1 protein may inhibit normal pronephros development. Consistent with this idea, microinjection of the *strabismus* mRNA, a member of the planar polarity genes, or morpholino antisense oligo into the *Xenopus* embryos resulted in the same trunk shorting phenotype due to inhibition of planar polarity signaling [40]. In embryos injected with an activator form of WT1 (VP16-WT1), expression of the pronephros marker was reduced (yellow arrows and arrowheads in Figure 4C,D), similar to wild-type *wt1* mRNA injection described above. Conversely, injection of a repressor form (En-WT1) mRNA expanded marker gene expression (blue arrows and arrowheads in Figure 4C,D). These results suggest that some of the WT1 target genes inhibit pronephros development. In other words, VP16-WT1 reduced the pronephros by activating the transcription of genes that inhibit pronephros development, and En-WT1 expanded the pronephros by suppressing the transcription of those genes (Figure 4C,D). In the future, it will be essential to analyze the functions of genes interacting with the WT1 and the WT1 target genes in the embryos to elucidate the molecular mechanism of the WT action in pronephros development.
